# Elucidation of interleukin-19 as a therapeutic target for breast cancer by computational analysis and experimental validation

**DOI:** 10.1016/j.sjbs.2023.103774

**Published:** 2023-08-11

**Authors:** Shazia sofi, Nusrat Jan, Hina Qayoom, Mustfa Alkhanani, Abdullah Almilaibary, Manzoor Ahmad Mir

**Affiliations:** a Department of Bioresources, School of Biological Sciences, University of Kashmir, Srinagar 190006, J&K, India; b Department of Biology, College of Science, Hafr Al Batin, University of Hafr Al-Batin, 31991, Saudi Arabia; c Department of Family and Community Medicine, Faculty of Medicine, Al Baha University, Albaha 65511, Saudi Arabia

**Keywords:** Upregulation, Prognosis, Breast cancer, Gene ontology, Overall survival, Pathogenesis

## Abstract

Interleukin 19 (IL-19) is a cytokine produced by monocytes and belongs to the family of IL-10. The IL-19 protein stimulates fibronectin (FN) expression and assembly, metastasis, and cell division in breast cancer (BC) cells. IL-19, which is connected to breast pathogenesis and has an autocrine action in BC cells, is a key predictor of prognosis for many tumour forms, including breast cancer. Augmented IL-19 expression has been related to poorer clinical outcomes for patients with BC and directly enhances proliferation and migration while also serving as a microenvironment for tumour formation. The main aim of our study was to examine the expression profile, functional role, and prognostic significance of interleukin-19 in BC pathogenesis and also to find out the molecular mechanism of IL-19 in BC. In this work, we used the various computational approach and tools, to evaluate the expression profile and prognostic implication of IL-19 in BC and discover the role of IL-19 in BC pathogenesis. IL-19 was shown to be highly upregulated in BC as compared to other interleukins. Also, its levels were highly overexpressed in liminal BC patients, mostly in 3rd stage groups under the age group of 21–40 years. IL-19 levels were increased in BC and elevated expression of IL-19 was examined to have worse overall survival (OS). The KEGG analysis and gene ontology of IL-19 depict that IL-19 is significantly augmented in cytokine activity and receptor-ligand activity and also in the JAK-STAT signaling pathway. Moreover, IL-19 showed a high correlation with IL20RA, as later is involved with the JAK-STAT signaling pathway. The *in-vivo and in-vitro studies* have also reflected that upregulation of IL-19 enhances tumor development and affects clinical outcomes in BC patients through several pathways including the JAK TAT signalling pathway. Overall, our study indicates that IL-19 increases tumour growth and that inhibiting it in addition to standard treatments will greatly improve BC patient’s therapeutic responses.

## Graphical abstract

**Figure:**
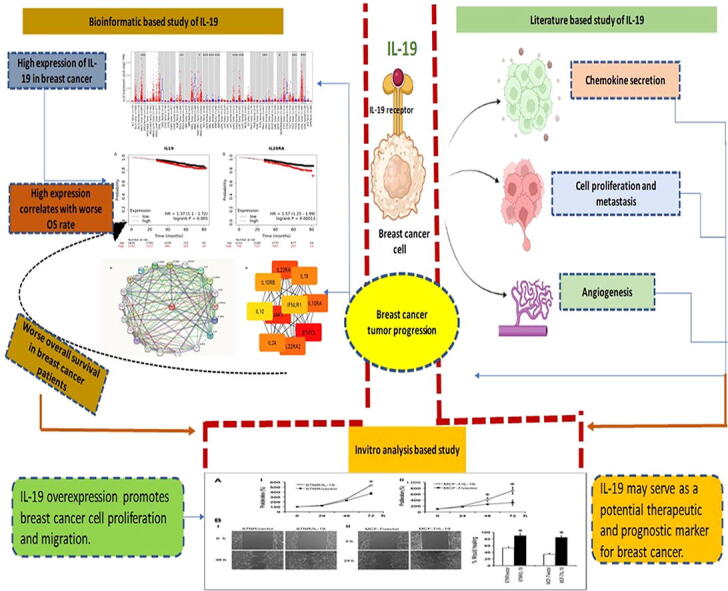

## 1 Introduction

Cancer is a highly complicated disease and is one of the main reasons behind the death rate throughout the globe. It is the 2nd main cause of mortality in the United States (US). An estimated 20 million new instances of cancer and 10 million cancer-related deaths occurred worldwide. Over the following two decades, the burden of cancer will rise by around 60%, placing additional strain on healthcare systems, individuals, and communities. By 2040, it is anticipated that there will be roughly 30 million additional instances of cancer worldwide, with the largest increases occurring in low- and middle-income nations (Zhang et al., 2023). Worldwide it remains a major health concern (Correia et al., 2021, Mir et al., 2022). According to GLOBOCAN 2020, around ten million deaths of cancer were recorded and almost 19.3 million new cases of cancer appeared in 2020 (Sung et al., 2021). Worldwide in women, BC is the most frequent type of cancer and it is the 2nd major reason of mortality in females (Sofi et al., 2022). Almost 1.67 million cases are diagnosed annually and about 1 in 37 women die due to BC. In a recent epidemiologic study, a 20% rise in the number of BC incidences has been reported globally resulting in 522,000 deaths since 2008 (Mir et al., 2020). According to GLOBOCAN 2020, the most often diagnosed cancer has been BC with 2.3 million fresh cases approximately followed by other types of cancer like colorectal cancer, lung cancer, prostate, and stomach cancer (Sung et al., 2021). BC is a heterogeneous disease and consists of three major tumor subtypes, which depend on the availability of molecular biomarkers for HER2, estrogen receptor (ER), or progesterone receptor (PR) (Mehraj et al., 2022). The subtypes of BC, which are categorized according to the PR, or ER expression and HER2 gene, have specific risk profiles and treatment plans (Lee et al., 2019). In BC, the accurate process of initiation is not known. Although for breast cancer initiation and progression, some hypothetical theories have emerged, which include the stochastic theory and cancer stem cell theory. cancer stem cell theory proposed that each tumor sub-types is initiated from the same stem cells or progenitor cells. Mutation in progenitor or stem cells results in different tumor phenotypes. The other theory called stochastic theory specifies that all tumor subtypes are derived from a single type of cell, which can be a progenitor cell or differentiated cell or stem cell. Breast cells can transform into tumor cell when sufficient random mutations have accumulated. Both the theories failed to explain fully the origin of BC in humans (Esquivel-Velázquez et al., 2015, Sun et al., 2017).

**Fig. 1:**
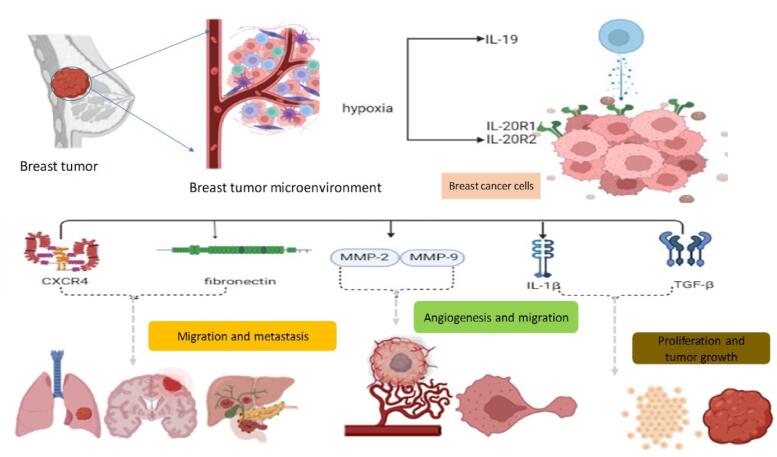
Working Mechanism of Interleukin 19 (IL-19) in breast cancer.

Inflammation is suggested as a vital participant in tumor initiation, angiogenesis, promotion, and metastasis (Greten and Grivennikov 2019). According to reports, inflammation has a role in the onset of a variety of diseases, including metabolic disorders, obesity, autoimmune conditions, and neoplastic conditions. The deadliest of these to affect people is cancer. It has been found that chronic inflammation plays a role in about 25% of cancer cases (Lan et al., 2021). A collection of useful proteins called cytokines is secreted by the immune system. Initially, they were referred to be immune response and inflammation modulators. However, a growing number of studies have discovered a link between elevated levels of certain cytokines and the development and spread of malignancies. These cytokines are part of a coordinated system that aids in or promotes the growth of cancer(Zamarron and Chen 2011). Several studies have associated cytokines with breast cancer development and related inflammation with breast cancer (BC) poor prognosis (Mehraj et al., n.d., Goldberg and Schwertfeger, 2010). Cytokines are soluble glycoproteins of low molecular weight (generally less than 30 kDa), secreted by the immune cells. In the TME, cytokines perform intercellular communication to favor cancer progression (Nicolini et al., 2006). Cytokines may be broadly grouped into the following protein families, which include colony-stimulating factors, interferons, and members of the tumor necrosis factor (TNF), interleukins, and chemokines (Esquivel-Velázquez et al., 2015). Increased level of cytokines is responsible for cancer development, drug resistance, and progression or metastasis within the tumor microenvironment (TME). Cytokines are the critical regulators of cell growth, development, differentiation, and migration and therefore cytokines notably affect the growth of tumors in vivo. They are immunomodulating agents and are required for immune regulation of both adaptive immune responses as well as innate immune responses. Under pathological and physiological conditions, these proteins and their receptors are produced in organisms (Nicolini et al., 2006). Some cytokines modulate the inflammatory TME. Chemokines and cytokines, being the inflammatory mediators in the TME, influence breast cancer (BC) development (Mehraj et al., 2022). Interleukins like IL-6, IL-1, and IL-11, trigger breast cancer (BC) development and invasion. It also activates intracellular signaling by nuclear factor kappa B (NF-κB) and cytokine receptor, stimulating tumor development. Several cytokines like interferons (IFNs), IL-12, and IL18 inhibit breast cancer (BC) proliferation and invasion (Hsing et al., 2012) **(**Table 1**)**. Another interleukin that maintains a microenvironment favorable for breast tumor progression is IL-19. In BC cells, interleukin IL-19 operates in an autocrine manner and stimulates the migration and proliferation, metastasis by up-regulating TGF-β, fibronectin, CXCR4, IL-6, IL-1β, MMP-9, and MMP-2 (Chen et al., 2013). Interleukin-19, a cytokine secreted by monocytes, in the interleukin-10 family, is expressed by several tumor cells which include invasive ductal carcinoma of the breast, squamous cell carcinoma (SCC) of the esophagus, tongue, lung, and skin. The IL-19 expression in BC tissue is linked with an increased mitotic rate leading to worse metastasis-free survival, disease-specific survival, high grade tumor metastasis, and worse prognosis (Chen et al., 2013). Recent studies conducted both *in-vitro* and *in-vivo*, have explored the IL-19 mechanism in BC cell lines of humans (Hs578T and MCF-7) and mice (4 T1 and 67NR) (Chen et al., 2013). IL-19 increases the expression level of all the factors like CXCR4, interleukin-6, TGF-β, IL-1β, MMP-2, MMP-9, and fibronectins that promote tumor development, migration of BC cells and is also linked with metastasis in breast cancer **(**Fig. 1**)** (Méndez-García et al., 2019).

**Table 1:** Characteristics of some significant Interleukins.

**S. N**	**Interleukin**	**Role**	**Expression**	**Synthesis**	**Prognosis**	**survival**	**Ref**
1	IL-6	Aid tissue takeover; promote EMT; induce angiogenesis; activate STAT3 pathway	High	T-cells, macrophages	Poor	Worse	7,12,14
2	IL-1	Proinflammatory cytokine, promotes EMT, angiogenesis, migration & bone metastasis	High	Monocytes, macrophages, endothelial cells, and epithelial cells	Poor	Not determined	7,12,14,22
3	IL-11	Promote tumorigenesis, proliferation, and angiogenesis and is associated with bone metastasis	High	Mesenchymal stem cells, fibroblast cells	Poor	worst	14,25,26
4	IL-10	Inhibits tumor growth mechanism by silencing IL-6 & metastasis; promotes tissue takeover & angiogenesis	High	Monocytes, B, and T cells	Poor	Not determined	7,14
5	IL-8	Promotes cancer development and metastasis	Low	Fibroblast cells, endothelial cells, neutrophils, macrophages	Poor	Increased risk of death	7,12,14
6	IL-19	Promotes Tumor, lung metastasis, development, migration & proliferation	High	Keratinocytes, monocytes, B-cells	Poor	worse	7,12,14,15
7	IL-20	Promotes metastasis, and proliferation by upregulating MMPs & cathepsins enhances tumor development	High	Keratinocytes	Poor	worse	7,12,14

IL-19 activates STAT3 by binding to a heteroreceptor (IL-20R1 and IL-20R2) and initiates cell proliferation (Hsing et al., 2012). High IL-19 expression helps in tumor development, proliferation, metastasis, and migration in breast cancer (BC), suggesting IL-19 as a prognostic marker/indicator. Il-19 is an important target in BC and to minimize the negative influences of IL-19, the use of anti-IL-19 monoclonal antibodies may help to improve therapeutic strategies (Mohammed 2022). IL-19 knockdown specimens showed inhibition in endogenous fibronectin expression and cancer cell migration. Also, IL19 inhibition inhibits hypoxia in BC 4 T1 cell lines (Hsing et al., 2012). In the existing study, we used a combined bioinformatic method to examine the functional role of IL-19 in BC as well as its expression profile. Using the freely available web tools, it was also possible to determine the relationship between IL19 and other genes as well. We also examined the association of IL-19 with tumor stroma in Bc. Here, we reveal that BC has significant dysregulation in IL-19 expression. Deregulated expression of IL-19 and IL20RA was also discovered to have an impact on BC patients' overall survival (OS). Enrichment studies revealed that IL-19 is essential for the function of JAK-STAT and cytokine receptor ligands and that regulating IL-19 in conjunction with established medications will be a potential strategy for treating BC patients. The studies have also revealed that IL-19 overexpression induces several pathways including JAK STAT pathway in 4 T1 BC cells.

Further, the in vitro studies on IL-19 done by Hsing et al., also throw light on the fact that IL-19 is a significant mediator in BC and is related to lymph node metastasis and distinct metastasis. To move further down the line, the in vivo studies done by Hsing et al., also strengthen our study that IL-19 overexpression enhanced bc development and lung metastasis. Additionally, IL-19 involved the role of MMP2, IL-6, MMP9, IL-1β, CXCR4, fibronectin, and TGF- β for tumor development. (Hsing et al., 2012).

## 2 Methodology

### 2.1 Expression pattern of IL19 in BC

The extensive online portal TIMER 2.0 (http://timer.cistrome.org/) is used to examine expression patterns and immune infiltrates in numerous types of cancer (Li et al., 2020). Using TCGA datasets, this online portal was used to examine the pattern of IL-19 expression in various malignancies. Additionally, GEPIA2 (http://gepia2.cancer-pku.cn/#analysis) assessed the expression profiles of IL-19 in several cancer types. Using a common processing pipeline, the extensive tool GEPIA2 analyses the RNA sequencing data of 9736 cancer patients and 8587 normal patients from the GTEx and TCGA projects (Tang et al., 2017).

### 2.2 UCSC XENA

UCSC XENA source was used to study the expression of different interleukins in BC, and the analysis produced a heatmap (https://xena.ucsc.edu/). Users can investigate functional genomic data sets using UCSC Xena to look for associations between phenotypic and/or genomic variables (Goldman et al., 2020). The database has got several datasets and data hubs and can be used for several other purposes like survival analysis etc.

### 2.3 UALCAN

An online portal for studying cancer OMICS data is UALCAN (https://ualcan.path.uab.edu/). Among other things, this database is made to make it easier to obtain freely accessible cancer OMICS data to identify biomarkers or validate potential candidate genes in silico (Chandrashekar et al., 2017). It is created on PERL-CGI and includes high-quality visualizations created using JavaScript. Users of the database have access to graphs and figures showing patient survival rates and gene expression patterns for families of genes that code for microRNAs, proteins, and lncRNAs. In this work, the expression of IL-19 was assessed in various tumor stages, patient sub-classes, and age groups, using the TCGA breast invasive cancer data.

### 2.4 Correlation analysis of interleukin 19

Utilizing a massive collection of functional association data, GeneMANIA (https://genemania.org/) discovers additional genes that are connected to a set of input genes. Protein and genetic relationships, pathways, co-expression, co-localization, and protein domain similarity are all examples of association data. We used the web resource GeneMANIA to forecast the role and relationship of IL19 in the gene-gene interaction (GGI) study. This online database is a flexible, and user-friendly portal for predicting the function of our genes/genes of interest and also gene sets (Warde-Farley et al., 2010). The database was used to predict the role and association of IL-19 with other significant genes.

### 2.5 KM plotter

KM plotter is an online portal that uses 25 k + samples from 21 tumor forms, especially BC, stomach, lung, and ovarian tumors, and allows users to develop the correlation between gene expression and survival (https://kmplot.com/) (Györffy et al., 2010). Some of the databases' sources include TCGA, EGA, and GEO. The basic aim of the tool is to make and verify survival biomarkers through *meta*-analysis. The OS of BC patients with IL19 was assessed here. There were two groups of BC patients, depending on the median expression of IL19: the higher expression and the lower expression cohort. Along with the HR, the association of IL19 and IL20RA expression and OS was examined.

### 2.6 Protein–protein interaction (PPI) analysis

Using STRING (http://stringdb.org) (v-11), a PPI network of IL19 was developed, with a confidence level of 0.7. STRING is a biological online portal designed to build and analyze functional associations between proteins (Szklarczyk et al., 2015). The PPI web was further investigated and envisaged using the Cytoscape application (v-3.8.2) (Smoot et al., 2011). The MCODE plug-in was used to get the PPI network's distinguished modules. The top ten HUB nodes in the PPI web were found employing the cytohubba plugin option (Chin et al., 2014).

### 2.7 IL-19 investigation in single-cell sequencing database

The IL-19 expression profile in primary and metastatic breast cancers was first processed using TISCH-2 (Tumor Immune Single-cell Hub) (https://tisch.comp-genomics.org/) (Sun et al., 2021). To ascertain the connection between IL19 expression patterns and tumour metastasis, we examined the expression of IL19 in original and metastatic BC datasets.

### 2.8 Pathway analysis and gene ontology

With the use of the Enrichr (https://maayanlab.cloud/Enrichr/), a Gene Ontology (GO) analysis of the IL-19 gene was completed. The molecular functions (MF), Cellular compartment, and Biological processes (BP) of gene ontology parameters for IL-19 were examined, and results with a p-value of 0.05 or higher were statistically significant. Additionally, using the Enrichr program, the KEGG pathways analysis of IL-19 was assessed.

## 3 Results

### 3.1 IL-19 is overexpressed in breast cancer

**Fig. 2:**
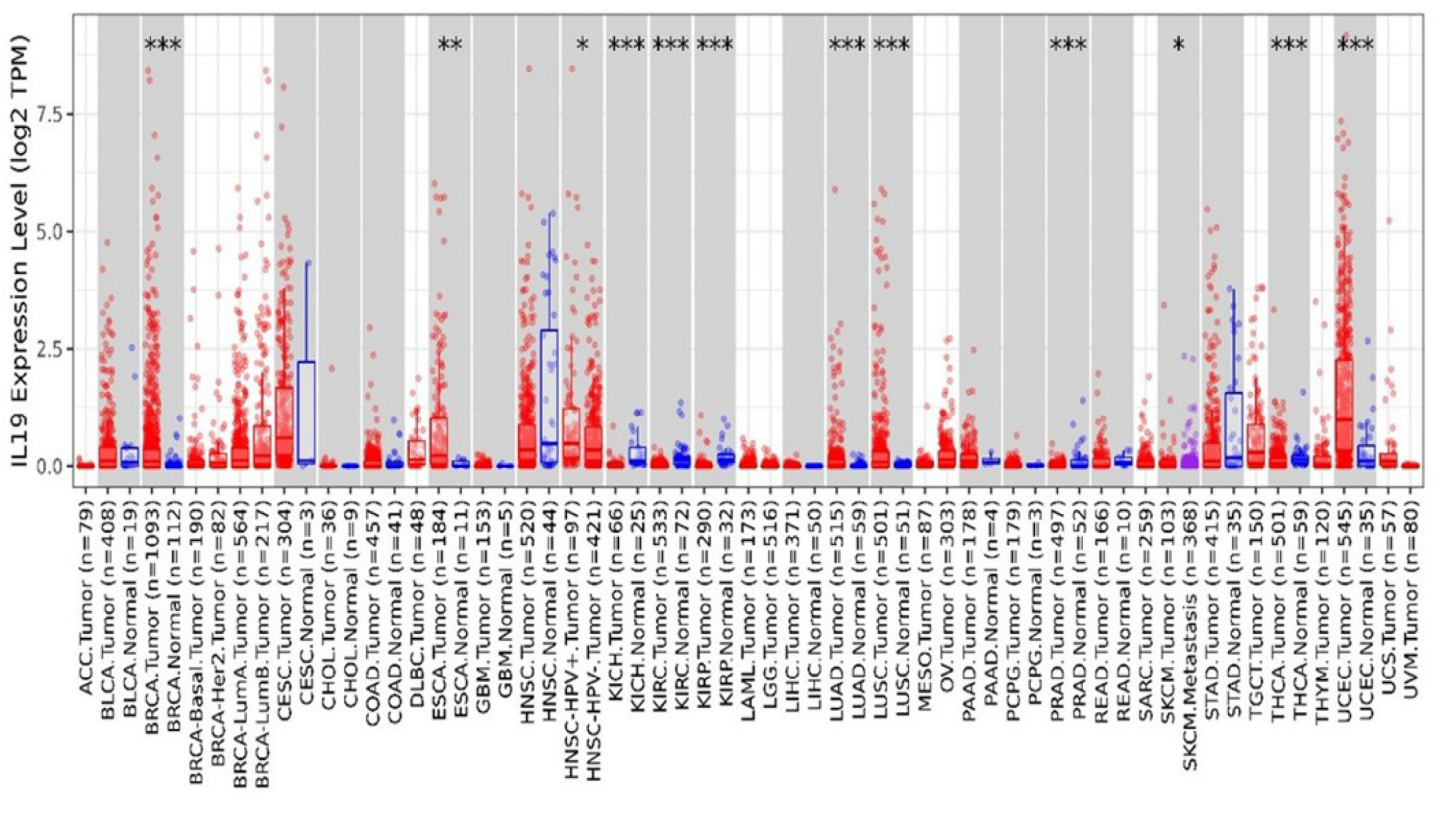
Differential expression of IL-19 in several cancers along with the normal ones. The box plots reveal that IL-19 levels are high in several cancers including breast cancer.

Using the TIMER 2.0 database, it was determined how IL-19 was expressed in various cancers. TIMER 2.0 analysis illustrates how numerous cancers have highly elevated IL-19 expression. The box plots revealed that IL-19 is upregulated in numerous cancers, including BC, BLCA, CESC, ESCA, HNSC, LUSC, OV, UCEC, STAD, LUAD, and UCS **(**Fig. 2**).**

**Fig. 3:**
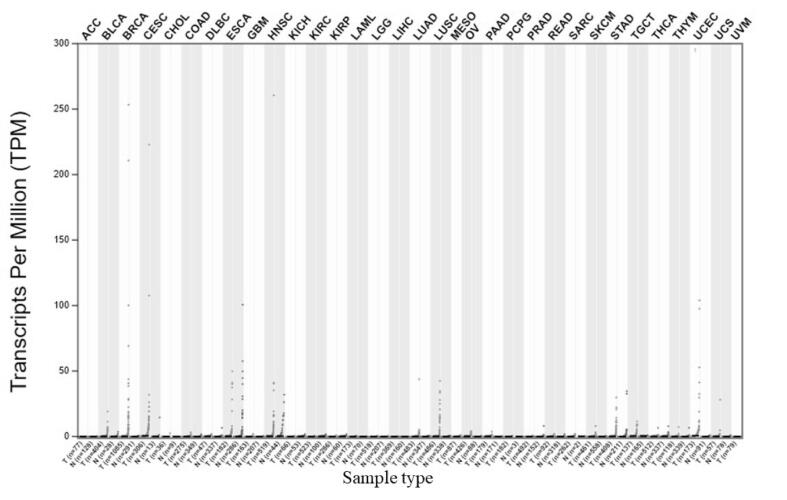
Expression profile of IL-19 in several cancers using GEPIA 2 database by LIMMA analysis. The expression of IL-19 in BC was upregulated along with other cancers.

The results were further confirmed using GEPIA 2 database. The expression profile of IL-19 in several cancers including BC was evaluated here. The results again revealed that IL-19 is upregulated in several cancers including BC, UCEC, and LUSC **(**Fig. 3**).**

### 3.2 IL-19 unlike other interleukins is overexpressed in BC

**Fig. 4:**
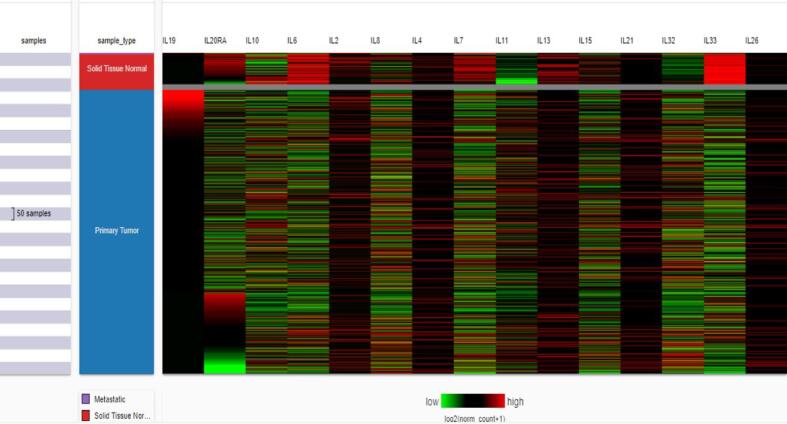
Heat map of different interleukins in breast cancer using UCSC XENA. Unlike other interleukins, IL-19 was highly upregulated in breast cancer patients.

After analyzing the expression profile of IL-19 in several cancers, we moved to develop a heat map of significant interleukins that depict a significant role in BC. A heat map of significant interleukins in BC was generated from UCSC Xena **(**Fig. 4**).**

The dataset used for developing the heatmap was TCGA BRCA. The results revealed by the heat map also put forth that IL-19 is highly upregulated in BC as compared to other interleukins. The most important interleukins that show upregulation include, IL-2, IL-19, and IL-20RA.

### 3.3 Expression pattern of IL-19 in various subclasses of BC

**Fig. 5:**
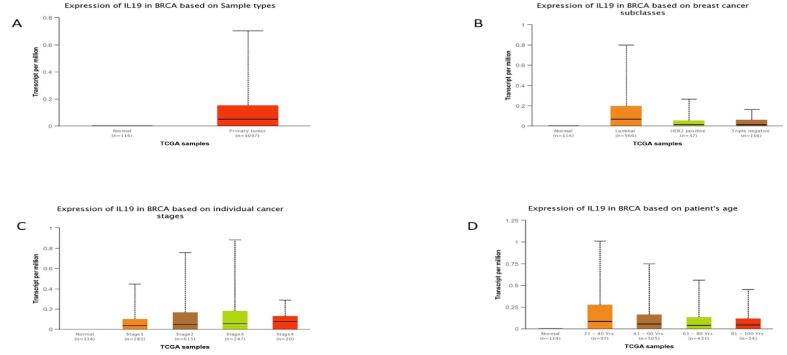
Expression profile of IL-19 in BC subclasses, age groups, and stages using the UALCAN analysis tool. (A) IL-19 levels were upregulated in BC tissues in comparison to normal ones. (B) Based on different classes of BC, Luminal BC patients expressed elevated levels of IL-19 in comparison to other BC subclasses. (C) Expression of IL-19 in different stages of BC, stage 3 showed higher levels of IL-19 as compared to other stages. (D) BC Patients between 21 and 40 years of age group showed higher expression of IL-19 when compared to other age groups of BC patients.

The expression profile of IL-19, based on the various parameters was evaluated using the UALCAN database. BC patients show significantly increased expression of IL-19 in comparison to normal patients **(**Fig. 5**A).** The expression profile of IL-19 among various BC subtypes was assessed and it was found that IL-19 was upregulated in luminal BC followed by TNBC and HER-2 positive tumors **(**Fig. 5**B).** Based on the cancer stage, the increased expression of IL-19 was observed in stage 3 followed by stage 2, stage 4, and stage 1 **(**Fig. 5 **C).** Additionally, it was discovered that patients with breast tumours in the age period of 21 to 40 and 41 to 60 years had overexpressed IL-19 as compared to the BC patients having the age group 81–100 years **(**Fig. 5 **D).**

### 3.4 Interleukin 19 highly correlates with IL10RA

**Fig. 6:**
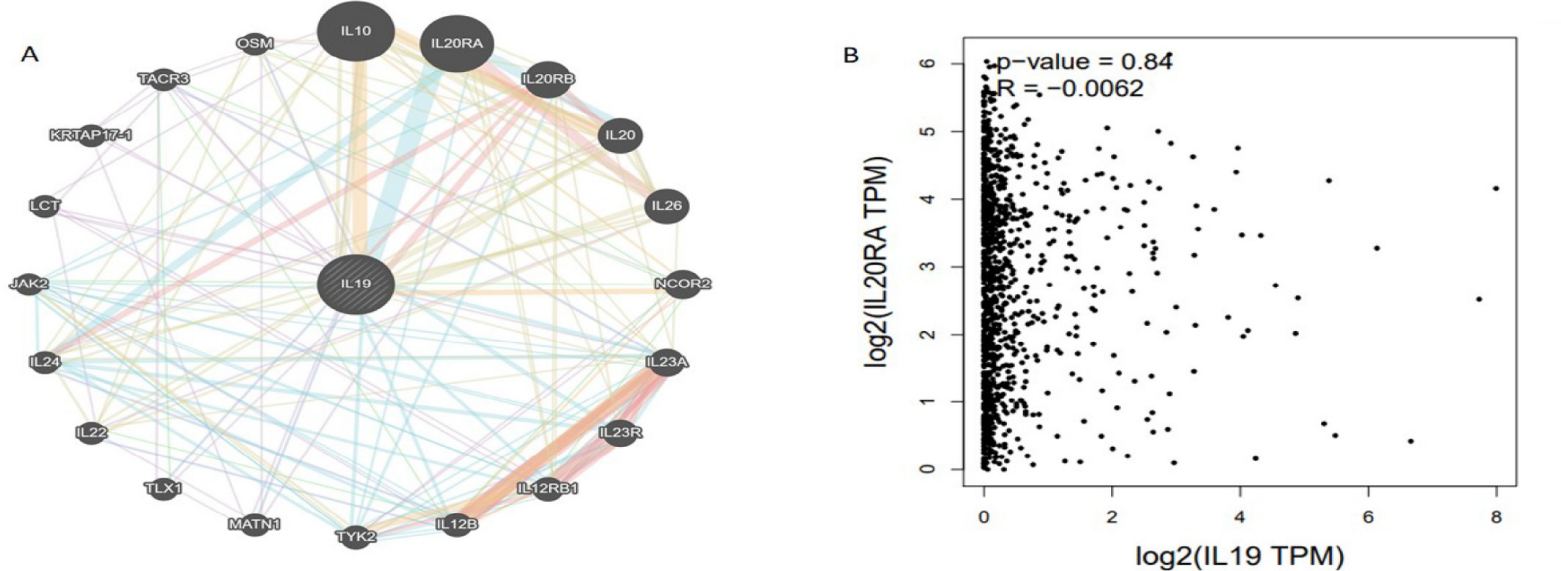
**A.** GGI network of IL-19 using GeneMANIA, IL-19 showed a high correlation with IL20RA followed by IL20RB, IL26, IL10, IL20, and several other genes. B. The Gepia2 results reflect a good association between IL and 19 and IL-20RA in BC with an r-value = 0.0062.

We studied the interactions between gene-gene network of IL-19 using the GeneMANIA portal. IL-19 revealed high interactions with IL20RA followed by IL20RB, IL26, IL10, IL20, and several other genes as shown in Fig. 6**A**. Physical relationships, co-localization, co-expression, genetic interaction pathways, and were the bases for the GGI network's interactions. The correlation between IL19 and IL20RA was examined using Gepia2 and the results depicted that IL19 and IL20RA extremely correlate in BC patients as R-value was 0.0062**.** Also, the interactions of IL19 and IL20RA were further intensely examined and the results confirmed that IL19 indirectly regulates the JAK-STAT and thereby leads to cell proliferation **(**Fig. 6**B).**

### 3.5 BC patients with upregulated IL-19 correlate with worse prognosis

**Fig. 7:**
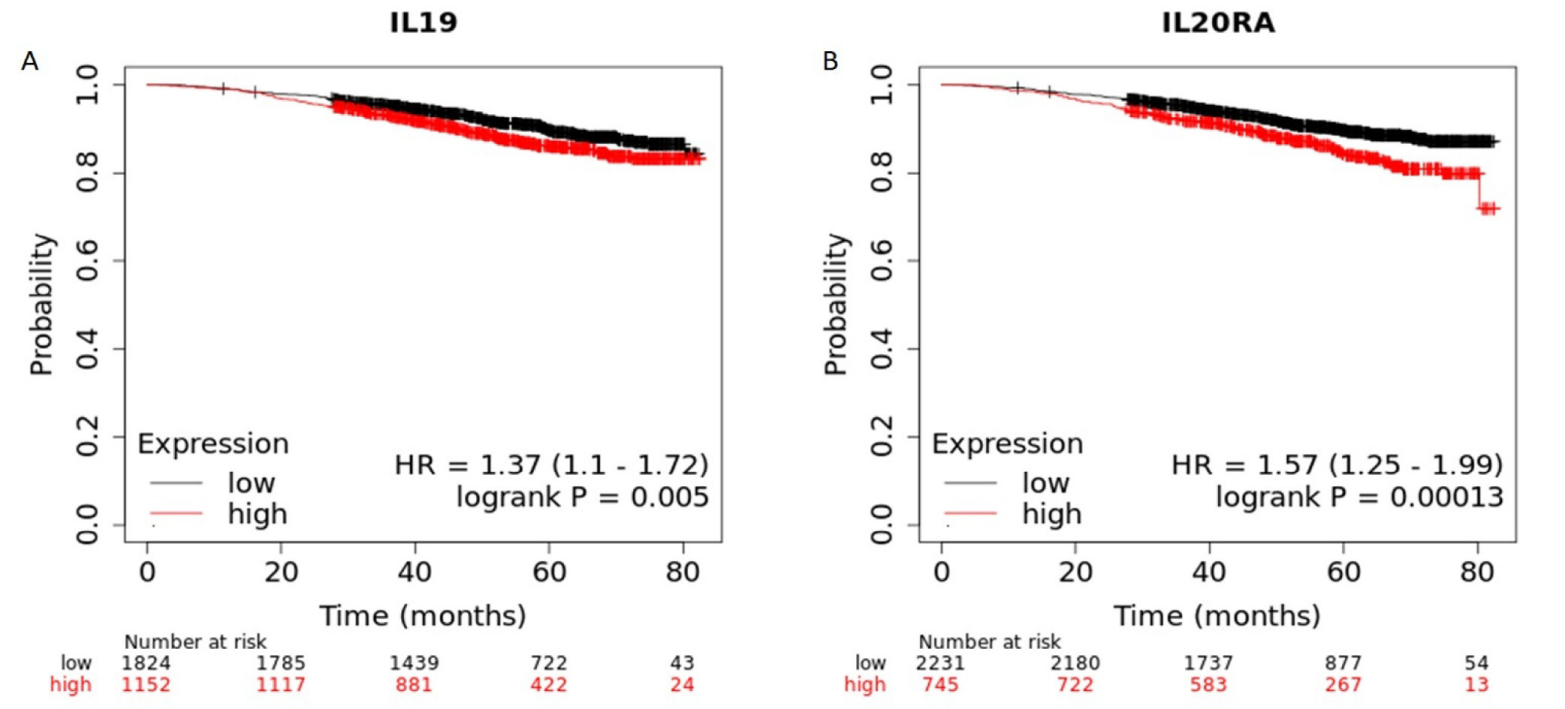
Kaplan Meir plotter analysis of IL-19 in BC patients. **A.** Decreased levels of IL-19 in BC correlate with high OS as compared to patients with higher levels of IL-19 (HR ​= ​1.37, log-rank P = 0.005). **B.** Increased expression of IL20RA associated with worse OS. (HR ​= ​1.57, log-rank P = 0.00013).

Kaplan Meir plotter was used to find the survival analysis of IL-19 in BC patients, based on overall survival (OS). The results depicted that reduced levels of IL-19 in BC correlate with high OS as compared to patients with elevated IL-19 mRNA levels (HR ​= ​1.37, log-rank P = 0.005) **(**Fig. 7**A).**

IL20RA, showing a higher association with IL-19 revealed a significant outcome on the OS of breast cancer patients. Greater expression of IL20RA allied with worse OS outcome. (HR ​= ​1.57, log-rank P = 0.00013) **(**Fig. 7**B).** Thus, higher levels of IL-19 and IL20RA in BC patients lead to worse prognosis as related to the patients having lower levels of IL-19 and IL20RA.

### 3.6 Protein-protein interaction (PPI) of IL-19

**Fig. 8:**
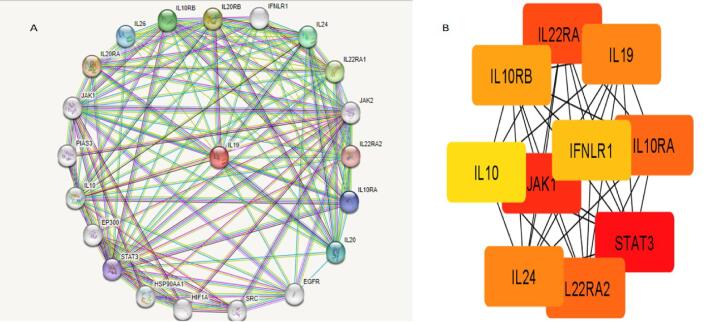
**A.** PPI of IL-19 **B.** Top 10 hub genes of the web were recognized using Cytohubba. The genes of the grid included IL-19, ILL22RA1, IL22RA2, IL10, IL10RA, IL10RB, IFNLR1, JAK1, STAT3 and IL24.

PPI was developed using STRING, by linking co-expressed 21 genes (nodes) with 128 proteins (edges). The PPI depicted an average local clustering coefficient: 0.812, PPI enrichment p-value of less than 1.0e-16, an average node degree: 12.2, and an expected edges: 41 (Fig. 8**A)**. The topmost 10 genes of the web, based on degree score value were recognized employing Cytohubba** (**Fig. 8**B)**. These top genes comprised IL-19, ILL22RA1, IL22RA2, IL10, IL10RA, IL10RB, IFNLR1, JAK1, STAT3 and IL24. MCODE plug-in option in the Cytoscape was analyzed to obtain the most crucial part of the PPI network.

### 3.7 Association of IL-19 with tumor stroma

**Fig. 9:**
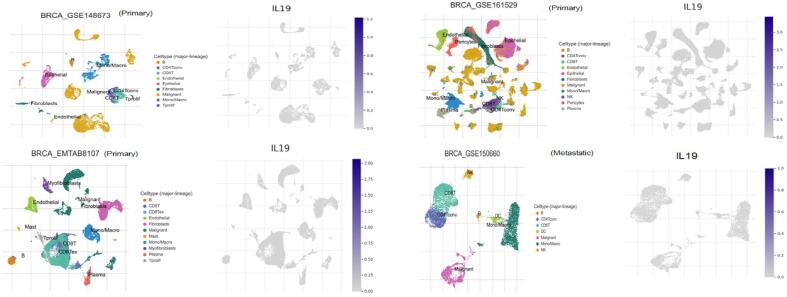
IL-19 expression showed a high expression in primary tumor as compared to metastatic cancers.

We analyzed the IL-19 expression profile in TISCH database. The expression profile analysis in few BC datasets having details about metastatic and primary tumors was performed. The study depicted that IL-19 is expressed in primary tumors as compared to metastatic tumors **(**Fig. 9**).** Moreover, IL-19 expression patterns showed heterogeneity across the primary tumor cell population. Furthermore, the immune cells, CD8T cells, B cells as well as malignant cells showed high IL-19 expression **(**Fig. 9**).**

### 3.8 Gene ontology and KEGG analysis

**Fig. 10:**
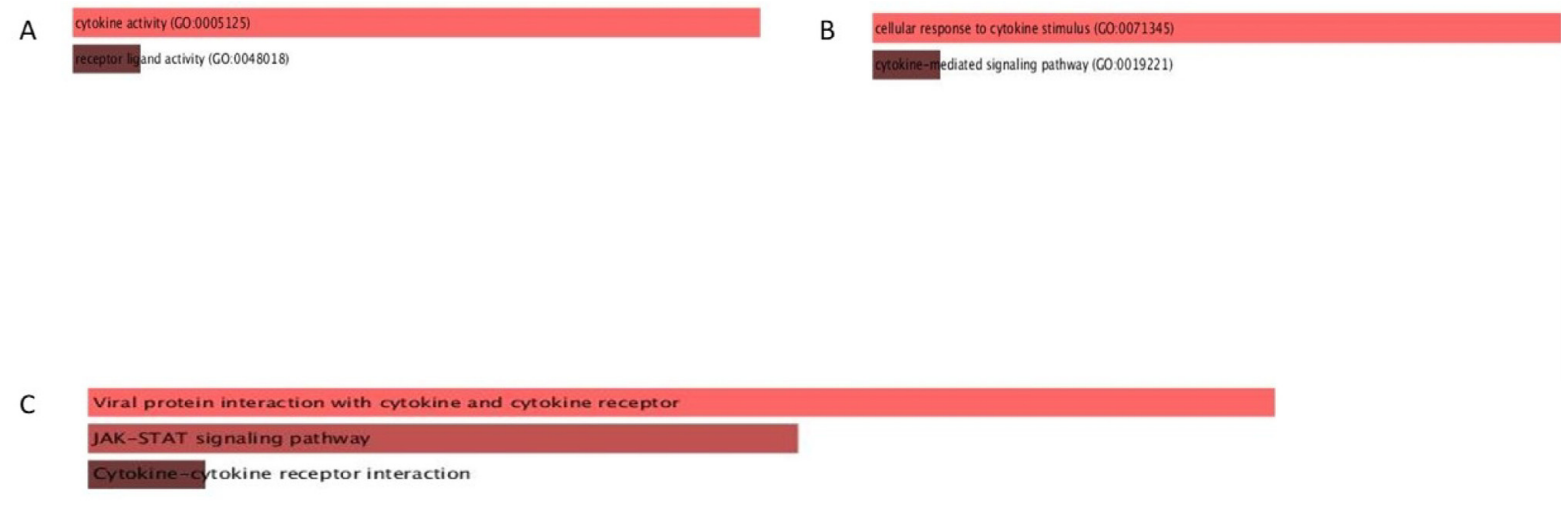
GO and KEGG pathway analysis of IL-19 using ENRICHR database. **A.** Among the MF, IL19 was involved in cytokine activity and receptor-ligand activity. **B**. Among the BP, IL-19 was found involved in cytokine-mediated signalling pathways and cellular response to cytokine stimulus**. C.** The KEGG analysis showed that IL19 has involved in JAK-STAT signalling, cytokine, and cytokine receptor association, and viral protein interaction with cytokines and their receptors.

The analysis of GO and KEGG analysis was done using the Enrichr database**.** Among the MF, IL19 was involved in cytokine activity and receptor-ligand activity (Fig. 10**A)**. Among the BP, IL-19 was found associated with the cellular response to cytokine stimulus and cytokine signaling pathways (Fig. 10**B**). The KEGG pathway study showed that IL19 is associated in viral protein association with cytokine and their receptors, JAK-STAT signaling, cytokine and cytokine receptor interaction as shown in Fig. 10**C.**

## 4 Discussion

In recent years, several experimental studies conducted both in-vivo and in-vitro have revealed the role of cytokines in cancer (Kany et al., 2019). From recent studies, it is evident and well-accepted that breast cancer (BC) development depends on an interaction between the stromal environment and tumor cells and not only on oncogenic modifications that occur within the epithelium (Feng et al., 2018). Also, inflammation is linked with poor prognosis and increased invasion (Greten and Grivennikov 2019). Cytokines like TNFα, IL-6, and IL-1β have been linked to poor progression and increased invasion. While some cytokines like IL-11, IL-1, TGFβ, and IL-6 induce breast cancer proliferation and invasion, on the other hand, some cytokines like interferons (IFNs), IL-18, and IL12 inhibit breast cancer (BC) growth and proliferation (Esquivel-Velázquez et al., 2015).

Recent experimental and clinical data shows interleukin-19 (IL-19) as a vital mediator in BC (Méndez-García et al., 2019). Elevated IL-19 expression in BC is linked with poor prognosis and lymph node metastasis. In BC, Il-19 has an autocrine action and offers an environment for tumor development, proliferation, migration, and metastasis. Therefore, inhibiting or antagonizing IL-19 in BC might have therapeutic potential (Hsing et al., 2012)**.**

In the current study, various bioinformatics tools have been applied to get the expression pattern of interleukin-19 in BC patients. For example, TIMER 2.0 and GEPIA were utilized to evaluate the expression profile of IL-19 in several types of cancers and the results revealed that IL-19 is highly upregulated in several cancers including breast cancer. Moreover, UALCAN was utilized to get the expression pattern of IL-19 in different subclasses and stages of breast cancer, and the results revealed that IL-19 is highly upregulated in the Luminal type of BC subtype, and the expression is upregulated in stage 3 of BC followed by stage 2. Further, the expression was found to be highly overexpressed in females within the age group of 21–40. Studies have also shown that in invasive ductal carcinoma specimens, increased IL-19 expression correlates with high tumor metastasis, advanced tumor grade, and worse survival (Hsing et al., 2012).

The study further led to analyze the survival analysis of IL-19 and its highly correlated gene IL20RA in BC. High expression of IL-19 in BC patients correlates with a worse OS rate, as the HR for poor OS was 1.37. Also, in BC patients, low IL20RA expression was found related to better OS and high expression showed worse OS, thus indicating that targeting IL-19 and IL-20RA is a hopeful treatment in BC. A study by Chung-Hsi Hsing et al; revealed that increased IL-19 enhances tumor development and affects Clinical Outcomes in BC (Hsing et al., 2012).

Additionally, the GGI and PPI networks demonstrated that IL-19 had a substantial relationship with cytokine ligand interaction pathways, indicating a key role for IL-19 in cancer malignancy. Further analysis of the PPI networks demonstrated that IL-19 may control JAK-STAT in BC both directly and indirectly. Moreover, IL-19 interacts with IL-20RA and activates JAK-STAT signaling. An earlier study has already confirmed that IL-19 interacts with IL-20RA and that IL20RA-triggered JAK1-STAT3 promotes the stemness of BC cells and that inhibiting IL20RA^+^ cells may be a potential strategy to minimize the stemness and reduce the drug-resistance of BC cells (Gao et al., 2021).

In addition, GO enrichment analysis depicted that IL-19 was strongly engaged in cytokine receptor ligand activity leading to cancer tumorigenesis. The potential of IL-19 to attach to IL-20RA suggests suggesting it might be useful in limiting the growth and spread of breast tumours. The study reported by Chung-Hsi Hsing showed that of the IDC specimens, Elevated tumour metastasis, advanced tumour stage, and poor survival were all linked to high IL-19 expression. Next, IL-19 was found pointedly augmented KEGG pathways for the viral protein interaction with cytokine and its receptors, and JAK-STAT signaling pathway. Regarding the IL-19 signaling pathways, the JAK-STAT pathway has been established to be accountable for BC progression. In addition to being linked to an increased mitotic rate, advanced tumour grade, and metastasis, IL-19 expression in BC tissue is also predicted to be worse for MFS and DSS with a risk that is more than three times higher (Esquivel-Velázquez et al., 2015).

**Fig. 11:**
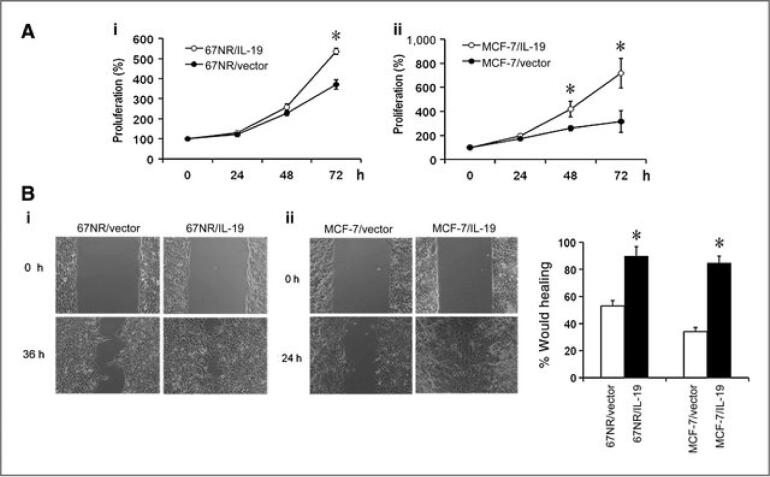
A) Overexpression of IL-19 enhances BC proliferation and migration. I) MCF-7/IL-19 and II) 67NR/IL-19 cell proliferation was significantly greater than that in control cells. B) Scratch wound healing assay. I) Cell migration in MCF-7/IL-19 and II) 67NR/IL-19 cells showed greater migration in scratch wound assay as compared to control vector cells (adapted from Hsing et al., (Hsing et al., 2012)).

Since interleukin-19 is highly deregulated in BC and its expression is related to the activation of JAK-STAT signaling pathways that ultimately leads to increased cell division and cell survival (Morris et al., 2018). In both human and mouse BC cells, treatment with IL-19 increases certain proliferation and migratory processes as well as fibronectin assembly and expression. IL-19 directly encourages proliferation in MCF-7 cells, which lengthens the time spent in the G2/M cell cycle phase (Huang et al., 2008). In 4 T1 cells having IL-19 knockdown, endogenous fibronectin expression, and tumour cell movement are confirmed, and treatment with IL-19 further boosts metastasis in IL-19 knockdown cells. Furthermore, BC cells are encouraged to proliferate, migrate, develop tumours, and metastasize when IL-19 is overexpressed **(**Fig. 11**)** (Hsing et al., 2012). The study done by Hsing et al., showed the role of IL-19 overexpression in lung metastasis of female BALB/c mice (Hsing et al., 2012). Based on this and the previous bioinformatics research, it is now possible to hypothesize that IL-19 might be a crucial prognostic and therapeutic biomarker for the detection, diagnosis, or prediction of prognosis in patients with BC.

## 5 Conclusion

In conclusion, our study depicted that BC patients had significant levels of IL-19 expression, which is linked to a worse OS. According to our findings, IL-19 may work as a potential therapeutic and prognostic marker for BC.

## 6 Declarations

### 6.1 Ethical approval

Ethical consent and approval of patients to participate is not applicable to our study.

### 6.2 Data availability

The BC datasets used in the study are openly assessed as https://portal.gdc.cancer.gov/projects/TCGA-BRCA and the survival data can be accessed as https://kmplot.com/analysis/index.php?p=service&cancer=breast.

Also, the databases like ULCAN, GEPIA, and TIMER have used R programming for analysis of datasets.

### 6.3 Date of data recovery

The datasets and the data used here have been recovered from their respective databases between the time 01-02-2023 to 1-03-2023.

### 6.4 Operating system used

The bioinformatic databases used in the study relied on LINUX-based operating and software systems.

### 6.5 Funding

This work has been funded by the JKST&IC (Jammu Kashmir Science Technology & Innovation Council Department of Science and Technology), India having grant No. JKST&IC/SRE/885–87.

## CRediT authorship contribution statement

**Shazia sofi:** Writing – original draft. **Nusrat Jan:** Writing – original draft. **Hina Qayoom:** . **Mustfa Alkhanani:** Writing – review & editing. **Abdullah Almilaibary:** Writing – review & editing. **Manzoor Ahmad Mir:** Conceptualization, Investigation, Supervision.

## Declaration of Competing Interest

The authors declare that they have no known competing financial interests or personal relationships that could have appeared to influence the work reported in this paper.
